# Betel-Quid Chewing, Heart Failure, and Premature Ventricular Contractions in Patients with Cardiopulmonary Symptoms

**DOI:** 10.3390/ijerph17207472

**Published:** 2020-10-14

**Authors:** Tien-Chi Huang, Wei-Tsung Wu, Ying-Chih Chen, Frances M. Yang, Wei-Chung Tsai, Chien-Hung Lee

**Affiliations:** 1 Division of Cardiology, Department of Internal Medicine, Kaohsiung Medical University Hospital, Kaohsiung Medical University, Kaohsiung 80756, Taiwan; 990314@gap.kmu.edu.tw (T.-C.H.); 990322@kmuh.org.tw (W.-T.W.); 2 Division of Cardiology, Ministry of Health and Welfare Hengchun Tourism Hospital, Pingtung 94641, Taiwan; 990329@kmuh.org.tw; 3 Department of Internal Medicine, Kaohsiung Municipal Siaogang Hospital, Kaohsiung Medical University, Kaohsiung 80756, Taiwan; 4 Department of Occupational Therapy Education, Medical Center, Kansas University, KS 66045, USA; fyang3@kumc.edu; 5 School of Medicine, College of Medicine, Kaohsiung Medical University, Kaohsiung 80708, Taiwan; 6 Department of Public Health, College of Health Science, Kaohsiung Medical University, Kaohsiung 80708, Taiwan; 7 Research Center for Environmental Medicine, Kaohsiung Medical University, Kaohsiung 80708, Taiwan

**Keywords:** areca nut, betel-quid, premature ventricular contraction, heart failure, substance use, cardiovascular disease

## Abstract

Betel-quid (BQ) is a commonly used psychoactive substance that renders a specific cardiotoxicity. The purpose of this study was to investigate the association between BQ chewing and premature ventricular contractions (PVC) in patients with cardiopulmonary symptoms, and examine the potential influences of cardiovascular and chronic diseases on such relationship. Participants were 146 patients with cardiopulmonary symptoms who participated in 24-h Holter electrocardiogram monitoring during 2012–2018 in a hospital serving residents that lived in a BQ high prevalence area. Data on substance uses and medical histories for cardiovascular and chronic diseases were collected. Baron–Kenny method was employed to evaluate possible mediation. In patients with cardiopulmonary symptoms, 36.3% were BQ users and 63.7% were nonusers. Adjusting for covariates, BQ chewing was significantly associated with heart failure and diabetes mellitus (adjusted odds ratio (aOR) = 3.4 and 2.3, respectively), but only heart failure was significantly correlated with a low and high level of PVC. Additionally controlling for the effect of heart failure, the risk of high PVC for BQ users reduced from 3.60 to 2.88; however, the risk for BQ chewers remained significant (95% CI: 1.06–7.84). Heart failure was found to explain 27.7% of the excessive effect of BQ use on high PVC. In conclusion, BQ use is directly associated with an elevated risk of high PVC in patients with cardiopulmonary symptoms. The higher risk might be elevated among patients who suffered heart failure. Given several research limitations, the findings from this study offer future opportunities for validation.

## 1. Introduction

Betel-quid (BQ), a mixed chewing product, is made by areca nut, often combined with a leaf of Piper betle smeared with white/red slaked limes, and occasionally tobacco or condiments [1]. Following caffeine, alcohol, and nicotine, BQ is the fourth psychoactive substance commonly consumed in the world [2,3]. This substance has been habitually consumed in six Asian populations with a prevalence of 9.8–43.6% in men and 1.8–46.8% in women [4]. In large-scale psychiatry studies, chronic BQ chewers were observed to have dependence symptoms and the related symptoms meet the *DSM*-5 criteria for a substance use disorder [5,6,7]. Epidemiological investigations have indicated that BQ users were at a higher risk of contracting systematic diseases that occurred in the brain, heart, lungs, gastrointestinal tracts, and reproductive organs [8,9,10]. The chewing of BQ can increase reactive oxygen species and arecoline *N*-oxide in oral cavity, which may elevate the risks of developing carcinomas of the upper aerodigestive tracts [11,12,13].

Clinical studies in chewers have found that BQ use can affect autonomic nervous function, increase heart rate and face temperature, and induce tachycardia and arrhythmias [14,15]. In cardiovascular investigations, BQ chewers were observed to have a higher risk for coronary artery disease and atrial fibrillation [16,17,18]. Additionally, the use of BQ was linked to non-obstructive acute coronary syndrome and out-of-hospital cardiac arrest among patients with severe ventricular arrhythmia [19,20].

Premature ventricular contraction (PVC) is a type of ventricular arrhythmia that induces abnormal heartbeats in lower heart chambers. This cardiac dysfunction has been closely linked to the development of cardiomyopathy, heart failure, and mortality from heart disease [21,22,23]. Prior studies have demonstrated that old age, male gender, body height, obesity, coronary heart disease, heart failure, myocardial infarction, hypertension, and physical inactivity are the risk factors for PVC [24]. Certain substance uses, such as tobacco smoking and alcohol drinking, have also been correlated with the development of PVC [24,25]. Given the intoxication of BQ in cardiac disorders, the relationship between this substance use and ventricular arrhythmias warrants a careful investigation.

In Taiwan, a large-scale epidemiological survey conducted in the southernmost township reported that residents in Mutan have a very high prevalence of BQ use (78.7% in females and 60.6% in males in 1997) [26]. Additionally, only two hospitals provide clinical treatment of heart disease for the inhabitants around Mutan. These unique epidemiological and geographic features constitute an appropriate condition to investigate the issue we raised. In this study, we monitored a patient sample with cardiopulmonary symptoms for ventricular arrhythmias using 24-h Holter electrocardiogram in a hospital that provides medical services to patients in Hengchun Peninsula, that includes Hengchun, Manzhou, Checheng, and Mutan townships and is the southernmost county in Taiwan. The purpose of this study was to investigate the association between BQ chewing and PVC in patients with cardiopulmonary symptoms, and examine the potential mediated influences of cardiovascular diseases (CVD) and chronic diseases on such relationship.

## 2. Materials and Methods

### 2.1. Participants

This is a hospital-based patient-series investigation carried out in one area hospital serving for Hengchun Peninsula inhabitants in southern Taiwan. There are about 50,000 people living on this Peninsula. Inhabitants who suffered cardiopulmonary symptoms often went to the research hospital or another hospital for treatments, because there is no large-scale medical center within a radius of 70–100 km of the Hengchun Peninsula. Study participants were the patients who came to the cardiovascular outpatient clinics requesting examinations and/or treatments for cardiopulmonary symptoms and agreed to participate in the measurement of 24-h Holter electrocardiogram. The cardiopulmonary symptoms experienced by study participants receiving a Holter monitoring included palpitation (51.4%), chest tightness (29.5%), short of breath (9.6%), and others (9.6%). This study was performed using a backtracking of medical records and secondary data analysis. Research protocol and the exemption of patient informed consent were approved by the Institutional Review Boards of Kaohsiung Medical University Hospital (IRB no., KMUHIRB-E(I)-20180319). During the period between 2012 and 2018, a total of 146 patients aged 35 years were included in this investigation.

### 2.2. Substance Use

The interviews with patients were performed by trained interviewers in hospital settings. Data on the use of BQ and cigarette, with regard to the amount daily consumed were collected and recorded in the medical records for each participant. BQ users were the patients who had chewed one or more quids of BQ per day for at least 1 year, and cigarette smokers were the patients who had smoked one or more cigarettes per day for at least 1 year. Substance nonusers were defined as those who never used the two types of substance or used them for less than 1 year.

### 2.3. Measurements of Holter Electrocardiogram and Echocardiography

The parameters recoded by a Holter electrocardiogram comprised maximum, minimum, and mean heart rates measured in a 24-h time period, sinus pause more than 1 s, and the amount, frequency, and duration of PVC and premature atrial contraction (PAC). The PVC burden was defined as the percentage of total beats that appeared PVC and the PAC burden was defined as the percentage of total beats that revealed PAC. Heart rate variability was also measured by the Holter electrocardiogram. However, data were available only for 54.1% of the participants (79/146). Echocardiography was performed for 76 patients (52.1%) because of clinical needs. The parameters measured in echocardiography included left atrium diameter, left ventricular diameter, interventricular septum, posterior wall septum of left ventricle, and left ventricular ejection fraction (LVEF).

### 2.4. Cardiovascular and Chronic Diseases

Medical information in regard to cardiovascular disorders, chronic diseases, and drug use were extracted from the measurements of biochemical laboratory, medical diagnosis records, prescribed medications, and echocardiography (if available) by the trained staff who were not aware of the results for substance use and Holter electrocardiogram. Medical histories collected for each participant included the initial date of diagnosis and continued treatments for heart failure, atrial fibrillation, coronary artery disease, stroke, hypertension, dyslipidemia, diabetes mellitus, and chronic kidney disease. The diagnosis of heart failure was according to Framingham criteria [27]. In those patients who fulfilled the criteria, they must also have at least one of the following diagnoses: (1) chest X-ray showed cardiomegaly (cardiothoracic ratio >0.5); (2) diuretics use included in current medication; or (3) left ventricular ejection fraction decreased to <0.5. Atrial fibrillation was one type of arrhythmia, which was characterized by the irregular and rapid beats of atrial chambers and determined either in Holter electrocardiogram or 12-lead electrocardiogram. The use of antiarrhythmic drugs, including β-blocker, calcium-channel blocker, digoxin, mexiletine (class Ib), propafenone (class Ic), and amiodarone (class III) was investigated for each patient. Coronary artery disease was defined as having the histories of myocardial infarction, angioplasty, receiving coronary artery bypass graft, or angina with aspirin use. Stroke was defined by a neurological deficit with an acute injury of the central nervous system, which is caused by a vascular cause. Hypertension was defined as having a history of hypertension (systolic blood pressure ≥140 mmHg or diastolic blood pressure ≥90 mmHg) or using any anti-hypertension drug. Dyslipidemia was defined as using any lipid-lowering agent including statins and/or fibrates. Diabetes was defined as using any oral anti-diabetic agent/insulin or having a laboratory data with HbA1c ≥ 6.5%. The symptoms of heart failure and diabetes were appeared after the use of BQ.

### 2.5. Statistical Analysis

Percentage and mean ± standard deviation were, respectively, used to express the distribution of categorical and continuous data, however median and interquartile range were employed for variables with an abnormal distribution. The differences in medians of heart rate, PVC-, and PAC-related parameters between BQ uses and nonusers were examined using Mann–Whitney test. Logistic regression models were employed to evaluate the association between binary outcome and study variables. As PVC burden is a zero-inflated distribution (i.e., having frequent zero-valued observations, 32.2% of participants in this study), we used the median (3.6 × 10^−4^) of PVC burden as a cutoff point and divided this parameter into three groups: no PVC, low PVC (PVC < median), and high PVC (PVC ≥ median) burden. Polytomous logistic regression models were employed to estimate adjusted odds ratio (aOR) of low and high PVC burden relative to no PVC, since three outcome groups were defined [28]. This type of logistic model can simultaneously assess the effect of explanatory variables on a dependent variable with >2 levels. The covariates that were controlling for all regression models included age, sex, and cigarette smoking.

**Figure 1:**
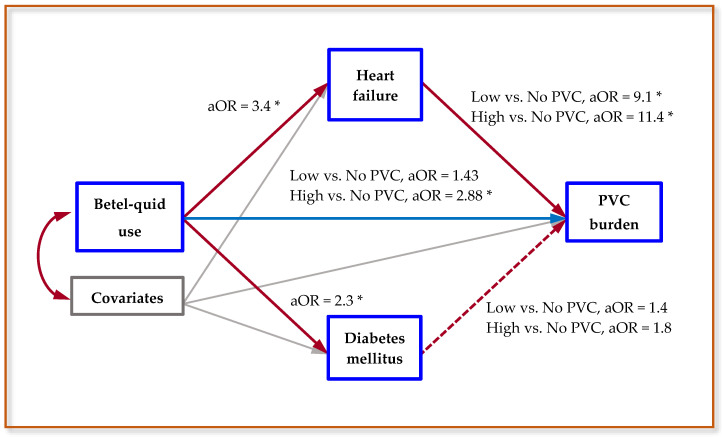
The schematic diagram of mediation analysis and summarized results between betel-quid use, heart failure, diabetes mellitus, and premature ventricular contractions (PVC) burden. **Note**: Covariates, including age, sex, and cigarette smoking, were adjusted for in all regression models. aOR denotes adjusted odds ratios, solid red lines represent a significant association, a dashed red line denotes a nonsignificant association, and a solid blue line reveals direct effect. * denotes *p* < 0.05.

In this investigation, all BQ users were a long-lasting chewer, cardiovascular and chronic disorders were preexisting or current diseases, and the PVC burden was the last measured parameter. We applied the Baron–Kenny technique to assess the possible mediated influences of cardiovascular and chronic diseases on the relationship between BQ use and abnormal PVC (Figure 1 presents the schematic diagram) [29,30]. This method has four requirements for a mediator, we applied it to our study as follows: (i) BQ use was significantly associated with specific cardiovascular/chronic diseases, (ii) specific cardiovascular/chronic diseases were significantly associated with abnormal PVC after adjusted for BQ use, (iii) BQ use was significantly associated with abnormal PVC, and (iv) the association between BQ use and abnormal PVC was higher than the same association after a specific mediator was controlled for. The excessive effect explained by an established mediator was calculated using the formula: [(Base model aOR − mediator-adjusted aOR)/(Base model aOR − 1)] × 100, where base model aOR was obtained from procedure (iii) and mediator-adjusted aOR was obtained from procedure (iv) [31,32].

## 3. Results

### 3.1. Demographic and Clinical Data

Table 1 presents the demographic and clinical factors associated with BQ use among Holter monitor patients, in those 36.3% were BQ users and 63.7% were nonusers. BQ chewers had a higher proportion of cigarette smoking (39.6%) than did the non-chewers (18.3%). There were no significant differences in the distribution of age, sex, the use of antiarrhythmic agent, and cardiopulmonary symptoms between BQ users and nonusers.

### 3.2. Electrocardiographic Parameters and BQ Use

Table 2 displays the electrocardiographic parameters in relation to BQ chewing among study participants. BQ users were observed to have a significantly more PVC and PVC burden than did the BQ nonusers (median: 28 vs. 2 for PVC and 2.7 × 10^−4^ vs. 0.2 × 10^−4^ for PVC burden). No substantial differences in medians for longest run, minimum/maximum heart rates, PAC, and PAC burden between the two BQ groups were found.

### 3.3. Cardiovascular/Chronic Diseases and BQ Use

Table 3 shows the aOR of cardiovascular and chronic diseases associated with BQ use among Holter monitor patients. In CVD, BQ chewers had a higher risk of heart failure, as compared with non-chewers (aOR = 3.4, 95% CI: 1.4–8.7). In chronic diseases, BQ use was correlated with a higher risk of diabetes mellitus (aOR = 2.3, 95% CI: 1.1–5.0). No significant relationship between the consumption of BQ and other cardiovascular and chronic diseases was found.

### 3.4. Cardiovascular/Chronic Diseases and Abnormal PVC

Table 4 presents the association of cardiovascular and chronic diseases with the level of abnormal PVC in patients with Holter monitoring. Controlling for covariates and BQ use, heart failure was significantly related with a low and high level of PVC burden in the measurements of Holter electrocardiogram (aOR = 9.1 and 11.4, respectively, both *p* < 0.05). However, the associations of diabetes mellitus with low and high levels of abnormal PVC burden were nonsignificant (aOR = 1.4 and 1.8, both *p* > 0.05).

### 3.5. AOR of Abnormal PVC and Excessive Effect Explained by Heart Failure

Table 5 displays the aOR of abnormal PVC burden and excessive effect explained by heart failure in study patients. Compared with those who did not chew BQ, patients who chewed BQ had a 3.60-fold higher risk of high PVC burden (95% CI: 1.36–9.51), with a 7.33-fold risk observed in chewers who used >20 BQ daily. In the polytomous logistic models that further adjusted for heart failure, both higher aORs were decreased. According to the rule of Baron–Kenny, heart failure can be regarded as a mediator of the association between BQ use and abnormal PVC. In Table 5, heart failure was observed to account for 27.7% of the relationship between BQ use and high PVC burden and 45.7% of the association between >20 BQ/day of use and high PVC burden. The risk of high PVC burden was still significant even when the effect of heart failure was controlled for (aOR = 2.88, 95% CI: 1.06-7.84). Figure 1 illustrates the relationships and summarized results between BQ use, heart failure, diabetes mellitus, and PVC burden.

### 3.6. Echocardiographic Parameters and Heart Rate Variability

As BQ chewing was related to a higher risk of heart failure, we further reviewed particular echocardiographic parameters and heart rate variability for patients who had the available data. As shown in Supplementary Table S1, no significant differences in left atrium diameter, left ventricular internal diameter, thickness of interventricular septum, and posterior wall in end-diastole and ejection fraction of left ventricle between BQ users and nonusers were found. BQ chewing was also not associated with the parameters measured for heart rate variability, as shown in Supplementary Table S2.

## 4. Discussion

This study presents findings to demonstrate that BQ use is associated with a higher risk of heart failure, diabetes mellitus, and PVC in patients with cardiopulmonary symptoms, and the relationship between BQ chewing and high level of PVC cannot be explained by heart failure. To the best of our knowledge, this is the first report that clarified the association across BQ use, heart failure, and PVCs using a 24-h Holter monitoring.

The association between CVD and BQ chewing has been reported in several studies [33,34]. In a prospective community cohort study, Yen et al. indicated that male BQ users had a 24% excess risk of developing CVD than did the nonusers [33]. Lan et al. found that BQ use was not only associated with total deaths but also with cardiovascular deaths [34]. However, a broad and relatively imprecise disease coding system (ICD-9-CM) was employed in both studies. This may limit the investigation of detailed dysfunctions for CVD. Using a precise disease diagnosis method validated by echocardiography and a 24-h Holter electrocardiographic monitoring for each patient, this study revealed the association between BQ use with PVCs and heart failure.

Studies have observed that, compared to chewing gum, BQ chewing increases heart rate, face temperature, tachycardia, and sympathetic dysmodulation with a gradual decrease in vagal modulation [14,15]. Chiang et al. in a two-patients of cardiotoxic case report revealed that the occurrence of paroxysmal supraventricular tachycardia and sudden death was linked to patients’ instant use of BQ [35]. Chou et al. in a letter comment indicated that such sudden death might be related to an acute ventricular fibrillation induced by a high level of BQ-derived arecoline and arecaidine exposure [20]. In a nationwide ecological study, a higher BQ use rate was found to be associated with a higher prevalence of atrial fibrillation in Taiwan [17]. PVC is a more common symptom in Holter electrocardiogram than atrial fibrillation and ventricular tachycardia. The PVC that can affect cardiac mortality is also a main clinical symptom of palpitation or chest discomfort in the clinical practices. In this study, we observed that BQ chewers had a higher risk of high PVC. These findings support the argument that BQ use may have a cardiotoxicity that affects arrhythmias and ventricular dysfunctions, and provide a significance to clinicians who treat patients with PVC.

In prior studies, frequent PVC has been associated with an increased risk for sudden/total cardiac death in general populations [36]. PVC more than 8% was reported to be correlated with a worsened global longitudinal strain [37]. Alternatively, patients with ≥24% of PVC burden were found to have a higher risk of impaired left ventricular systolic dysfunction [38]. Compared to the lowest quartile of PVC burden (0%–0.002%), patients having the highest quartile of PVCs burden (0.123%–17.7%) were identified to have a higher risk of decreased LVEF, congestive heart failure, and mortality [39]. BQ is an addictive substance that can induce BQ use disorder [7]. In this investigation, BQ chewing revealed a significant effect on high level of PVC. This implies that BQ use is a non-ignorable risk factor for PVC, given its addictive characteristic and cardiotoxicity.

Experimental studies have found that the nitrosated derivatives of areca nut alkaloids include a ring-shaped moiety that in configuration is similar to glucose, implying a possibility that BQ use might be diabetogenic [2]. In CD1 mice, areca nut intake was observed to induce glucose intolerance in adult mice and their F1 and F2 offspring [40]. One population-based cross-sectional study demonstrated a dose–response relationship of BQ use with hyperglycemia and diabetes, suggesting BQ might have a direct toxic to pancreatic beta cells [41].

In a large-scale population-based investigation, in that numerous cardiometabolic risk factors have been controlled, BQ users were observed to have a 1.3-fold risk of type 2 diabetes [41]. Similarly, people who used BQ for >10 years were found to have a 7.7-fold diabetes-and-hypertension-adjusted risk of coronary artery disease [18], and those who chewed >20/day of BQ had a 7.5-fold risk of obstructive coronary artery disease [16]. In one cardiovascular study, obstructive and non-obstructive ST-elevation myocardial infarction were both correlated with cardiotoxicity derived from BQ [19]. In cardiometabolic investigations, BQ use has been linked to the development of hypertension, hypertriglyceridemia, hyperglycemia, and metabolic syndrome [41,42,43]. These are all risk factors for type 2 diabetes and heart failure. In this study, we identified a positive association of BQ chewing with diabetes and heart failure, but the possibility that such relationship was mediated via these BQ-related risk factors cannot be excluded. Although our results tend to support the association of BQ use with diabetes and heart failure, the observed associations still cannot be precluded from other factors that were concurrently associated with BQ use and these two diseases.

Sympathetic activity can bring about heart rate increasing, systemic vasoconstriction, and enlarged venous tone, then increase ventricular preload and systemic vascular resistance; in response to these, the heart increases ventricular end-diastolic volume and stroke volume [44]. Chronic sympathetic stimulation can induce cardiac myocyte enlargement, increase myocardial mass, lead to enlargement of the left ventricular chamber, and finally bring about heart failure [45]. As BQ increased sympathetic activity [14,15], this provides a biomedicine mechanism to support the relationship between BQ use and heart failure.

In one follow-up study with 102 heart failure patients, 30.4% of patients were observed to have 101–1000 PVCs/24-h and 48% of patients had >1000 PVCs/24-h [46]. Electrolyte imbalance, increased sympathetic activity, and proarrhythmic effects of medications used for heart failure were considered the reason contributing to more PVCs in patients with heart failure [47]. In this investigation, heart failure was a preexisting disease that occurs prior to the measurement of Holter electrocardiogram. Thus, this condition interprets our findings in that 27.7% of the association between BQ use and high level of PVC was explained by heart failure. These findings offer an insight that BQ use might through multiple mechanisms elevate the risk of more PVCs among heart failure patients. If this viewpoint has been confirmed by other studies, BQ should be abstained from patients who suffered heart failure.

There are several limitations in this study. First, the cross-sectional nature of this investigation prevents a causal–effect inference about all conclusions. Second, the restricted sample size impedes a detailed evaluation for the relationship across BQ, heart failure, diabetes, and abnormal PVC. The limited samples were also difficult to use to differentiate the effects of other parameters that may link to these study variables. Third, certain cardiometabolic factors, such as risk components for metabolic syndrome, were not collected, which could confound or mediate the specific relationship between BQ use and PVC. Fourth, the type and duration of BQ used by the patients were not available, which prevent a full investigation for BQ characteristics consumed. This study has several advantages. First, all diagnoses for heart failure were confirmed cases and validated by an independent cardiologist who had no knowledge of the study results. Second, high BQ prevalence in study sample improved the power to clarify the study question for BQ consumption. Last, this is the first investigation that used a 24-h Holter electrocardiographic monitoring to examine the association between BQ use and PVC.

## 5. Conclusions

In patients with cardiopulmonary symptoms, our study provides an understanding about the direct association of BQ use with an elevated risk of high level of PVC due to its cardiotoxicity. The risk might be elevated through multiple medications among patients who suffered heart failure. However, given a limited sample size in this investigation, the findings from this study offer future opportunities for validation.

## Figures and Tables

**Table 1:** Demographic and clinical characteristics associated with betel-quid (BQ) use among Holter monitor patients.

	BQ Users		BQ Nonusers		*p* Value
Characteristics	No.	(%)	No.	(%)	
**Participants**	53	(36.3)	93	(63.7)	
**Age (mean ± SD), year**	67.5 ± 12.4		63.3 ± 14.3		0.075
35–49	5	(9.4)	19	(20.4)	
50–64	16	(30.2)	34	(36.6)	
65–74	12	(22.6)	15	(16.1)	
≥75	20	(37.7)	25	(26.9)	
**Sex, male**	29	(54.7)	39	(41.9)	0.137
**Cigarette smoking, yes**	21	(39.6)	17	(18.3)	0.005
**Use of antiarrhythmic agent ^1^, yes**					
β-blocker	18	(34.0)	20	(21.7)	0.107
Calcium-channel blocker	1	(1.9)	1	(1.1)	0.596
Digoxin	1	(1.9)	0	(0.0)	0.363
Class I/III drugs	0	(0.0)	5	(5.4)	0.101
**Symptom for Holter monitor user**					0.076
Palpitation	22	(41.5)	53	(57.0)	
Chest tightness	15	(28.3)	28	(30.1)	
Short of breath	8	(15.1)	6	(6.5)	
Others	8	(15.1)	6	(6.5)	

^1^ Class I/III drugs included mexiletine (class Ib), propafenone (class Ic), and amiodarone (class III).

**Table 2:** Electrocardiographic parameters associated with betel-quid (BQ) use among Holter monitor patients.

	BQ Users		BQ Nonusers		*p* Value
Parameters	Median	Q1–Q3	Median	Q1–Q3	
**Heart rate, beats/min.**					
Mean	75	68–85	73	65–81	0.746
Minimum	50	44–58	49	45–55	0.669
Maximum	116	104–136	120	102–131	0.509
**PVC, beats/day**	28	1–293	2	0–38	0.034
Pairs	0	0–1	0	0	0.577
Runs	0	0–0	0	0	1.000
Longest run	0	0–0	0	0	1.000
**PVC burden, 10^−4^**	2.7	0.1–22.4	0.2	0–3.8	0.016
**PAC, beats/day**	16	2.5–65.5	15	4–64	0.772
Pairs	0	0–2	0	0–1	0.947
Runs	0	0–1	0	0–1	0.985
Longest run	0	0–4	0	0–5	0.996
**PAC burden, 10^−4^**	1.4	0.2–6.9	1.5	0.4–8.5	0.772

Q1, 25th percentile; Q3, 75th percentile; PVC, premature ventricular contraction; PAC, premature atrial contraction.

**Table 3:** Adjusted odds ratio (aOR) of cardiovascular and chronic disease associated with betel-quid (BQ) use among Holter monitor patients.

Disease Outcomes	BQ Users (*n* = 53)		BQ Nonusers (*n* = 93)				
	No.	(%)	No.	(%)	aOR ^1^	(95% CI)	*p* Value
**Cardiovascular disease, yes**							
Heart failure	17	(32.1)	11	(11.8)	3.4	(1.4–8.7)	0.009
Atrial fibrillation	11	(20.8)	22	(23.7)	0.9	(0.4–2.2)	0.842
Coronary artery disease	3	(5.7)	5	(5.4)	0.9	(0.2–4.2)	0.874
Stroke	4	(7.6)	8	(8.6)	1.0	(0.3–3.6)	0.963
**Chronic disease, yes**							
Hypertension	41	(77.4)	62	(66.7)	1.4	(0.6–3.4)	0.419
Dyslipidemia	20	(37.7)	32	(34.4)	1.2	(0.6–2.5)	0.647
Diabetes mellitus	22	(41.5)	22	(23.7)	2.3	(1.1–5.0)	0.034
Chronic kidney disease	10	(18.9)	9	(9.7)	1.8	(0.6–5.1)	0.281

^1^ Participants with nonspecific disease investigated were treated as the reference group. All aORs were obtained from multivariable logistic regression model adjusted for age, sex, and cigarette smoking.

**Table 4:** Adjusted odds ratio (aOR) of premature ventricular contractions (PVC) associated with cardiovascular and chronic diseases among Holter monitor patients.

Diseases	No PVC	Low PVC Burden			High PVC Burden		
	No.	No.	aOR ^1^	(95% CI)	No.	aOR ^1^	(95% CI)
**Cardiovascular disease, yes/no**							
Heart failure	1/46	11/38	9.1	(1.1–77.3)	16/34	11.4	(1.4–95.4)
Atrial fibrillation	10/37	11/38	1.0	(0.3–2.9)	12/38	1.0	(0.3–2.9)
Coronary artery disease	1/46	3/46	3.2	(0.3–34.2)	4/46	4.1	(0.4–43.1)
Stroke	4/43	4/45	0.7	(0.1–3.2)	4/46	0.6	(0.1–2.8)
**Chronic disease, yes/no**							
Hypertension	33/14	33/16	0.4	(0.2–1.2)	37/13	0.5	(0.2–1.4)
Dyslipidemia	16/31	19/30	1.0	(0.4–2.4)	17/33	0.7	(0.3–1.8)
Diabetes mellitus	9/38	15/34	1.4	(0.5–3.8)	20/30	1.8	(0.7–4.9)
Chronic kidney disease	7/40	6/43	0.4	(0.1–1.4)	6/44	0.3	(0.1–1.0)

^1^ aORs were obtained from polytomous logistic regression models (outcomes: no, low, and high PVCs burden) adjusted for age, sex, and cigarette smoking, as well as betel-quid use.

**Table 5:** Adjusted odds ratio (aOR) of premature ventricular contractions (PVC) burden and excessive effect explained (EEE) by heart failure (HF) among Holter monitor patients.

	Base Model ^1^				HF-Adjusted Model ^2^					
Betel-Quid	Low vs. No PVC Burden		High vs. No PVC Burden		Low vs. No PVC Burden			High vs. No PVC Burden		
	aOR	(95% CI)	aOR	(95% CI)	aOR	(95% CI)	EEE ^3^	aOR	(95% CI)	EEE^3^
**Nonusers**	1.00	Ref.	1.00	Ref.	1.00	Ref.		1.00	Ref.	
**Users**	1.72	(0.65–4.61)	3.60 *	(1.36–9.51)	1.43	(0.52–3.94)	NA	2.88 *	(1.06–7.84)	27.7%
1–20 quids/day	1.94	(0.51–7.33)	2.85	(0.74–10.94)	1.54	(0.38–6.16)	NA	1.96	(0.46–8.26)	NA
>20 quids/day	1.58	(0.19–13.34)	7.33 *	(1.10–49.03)	1.15	(0.13–10.24)	NA	4.44	(0.63–31.48)	45.7%

NA, non-applicable due to nonsignificant excessive risk; Ref, reference; *, *p* < 0.05. ^1^ Base models were obtained from polytomous logistic regression models (outcomes: no, low, and high PVCs burden) adjusted for age, sex, and cigarette smoking (patients without PVC were the reference group). ^2^ Base models were additionally adjusted for heart failure (patients with no PVC were the reference group). ^3^ EEE (%) = [(Base model aOR − HF-adjusted aOR)/(Base model aOR − 1)] × 100.

## Supplementary Materials

The following are available online at https://www.mdpi.com/1660-4601/17/20/7472/s1, Table S1: Electrocardiographic characteristics associated with betel-quid use among Holter monitor patients, Table S2: Parameters of heart rate variability associated with betel-quid use among Holter monitor patients.
