# Supplementary material for: Betel-Quid Chewing, Heart Failure, and Premature Ventricular Contractions in Patients with Cardiopulmonary Symptoms
Source: Int J Environ Res Public Health. 2020 Oct 14;17(20):7472. doi: 10.3390/ijerph17207472 (PMC7602392; doi:10.3390/ijerph17207472)
Supplement: Supplementary file 1 [file ijerph-17-07472-s001.pdf]

Supplement Table S1. Electrocardiographic characteristics associated with betel-quid use among Holter monitor patients [1].

Parameters	BQ users (*n* = 29) (mean ± SD)	BQ nonusers (*n* = 45) (mean ± SD)	*p* value
LA (cm)	3.89 ± 0.91	3.92 ± 1.03	0.890
LVIDd (cm)	4.89 ± 0.96	5.13 ± 0.62	0.205
IVSd (cm)	1.44 ± 1.67	1.27 ± 1.39	0.655
LVPWd (cm)	1.09 ± 0.36	1.29 ± 1.38	0.454
EF (%)	65.46 ± 14.44	64.80 ± 11.44	0.828

LA, left atrium diameter; LVIDd, left ventricular internal diameter in end-diastole; IVSd, interventricular septum thickness in end-diastole; LVPWd, left ventricular posterior wall thickness in end-diastole; EF, ejection fraction of left ventricle; SD, standard deviation. [1]Only 76 patents were measured for these electrocardiographic parameters.

Supplement Table S2. Parameters of heart rate variability associated with betel-quid use among Holter monitor patients[1].

Parameters	BQ users (n = 31)		BQ nonusers (n = 48)		p value
	Median	Q1–Q3	Median	Q1–Q3	
SDNN, ms	117.60	81.79–165.28	130.51	97.05–161.33	0.520
RMSSD, ms	23.31	14.77–60.21	26.82	16.14–41.94	0.968
NN50, ms	3286	731–13972	3493	882–11165	0.920
pNN50, %	3.08	0.70–12.28	4.17	0.91–10.66	0.790
VLF, ms^2	3095	1972–4812	2777	2009–3747	0.366
LF, ms^2	3576	2009–7801	2167	1416–4369	0.100
HF, ms^2	1643	638–4596	1003	595–2150	0.148
Normalized LF, %	69.91	55.69–75.59	68.93	60.93–74.72	0.810
Normalized HF, %	30.10	24.41–44.31	31.07	25.38–39.07	0.810
LF/HF	2.32	1.26–3.10	2.22	1.56–2.94	0.810

HF, high frequency; LF, low frequency; NN50, the number of pairs of successive NNs that differ by more than 50 ms; pNN50, the proportion of NN50 divided by total number of NNs; Q1, 25th percentile; Q3, 75th percentile; RMSSD, root mean square of successive differences; SDNN, the standard deviation of NN intervals; VLF, very low frequency. [1]Only 79 patents were measured for these heart rate variability parameters.
